# Salicylic acid is a key player of Arabidopsis autophagy mutant susceptibility to the necrotrophic bacterium *Dickeya dadantii*

**DOI:** 10.1038/s41598-021-83067-6

**Published:** 2021-02-11

**Authors:** Martine Rigault, Sylvie Citerne, Céline Masclaux-Daubresse, Alia Dellagi

**Affiliations:** Institut Jean-Pierre Bourgin, UMR1318 INRA-AgroParisTech, INRAE Centre de Versailles-Grignon, Université Paris-Saclay, Route de St Cyr (RD 10), 78000 Versailles Cedex, France

**Keywords:** Plant sciences, Plant immunity, Virulence

## Abstract

Autophagy is a ubiquitous vesicular process for protein and organelle recycling in eukaryotes. In plant, autophagy is reported to play pivotal roles in nutrient recycling, adaptation to biotic and abiotic stresses. The role of autophagy in plant immunity remains poorly understood. Several reports showed enhanced susceptibility of different Arabidopsis autophagy mutants (*atg*) to necrotrophic fungal pathogens. Interaction of necrotrophic bacterial pathogens with autophagy is overlooked. We then investigated such interaction by inoculating the necrotrophic enterobacterium *Dickeya dadantii* in leaves of the *atg2* and *atg5* mutants and an *ATG8a* overexpressing line. Overexpressing *ATG8a* enhances plant tolerance to *D. dadantii*. While *atg5* mutant displayed similar susceptibility to the WT, the *atg2* mutant exhibited accelerated leaf senescence and enhanced susceptibility upon infection. Both phenotypes were reversed when the *sid2* mutation, abolishing SA signaling, was introduced in the *atg2* mutant. High levels of SA signaling in *atg2* mutant resulted in repression of the jasmonic acid (JA) defense pathway known to limit *D. dadantii* progression in *A. thaliana*. We provide evidence that in *atg2* mutant, the disturbed hormonal balance leading to higher SA signaling is the main factor causing increased susceptibility to the *D. dadantii* necrotroph by repressing the JA pathway and accelerating developmental senescence.

## Introduction

Being sessile organisms, plants cannot escape the multitude of stresses they are exposed to. They can be attacked by microbial pathogens and have the ability to protect themselves by the deployment of complex defense mechanisms. Defense-related responses involve protein phosphorylation, accumulation of reactive oxygen species (ROS), ionic fluxes and biosynthesis of phytohormones leading to transcriptional activation of genes coding for enzymes involved in the synthesis of antimicrobial compounds such as phytoalexins or pathogenesis related (PR) proteins^1–6^. To activate these defenses, plants are equipped with receptors that can detect different types of molecules either derived from the pathogen or derived from their own tissues^7–9^. Hormones are strongly involved in plant signaling during pathogen attack^10–13^. Depending on the lifestyle of the pathogen, different hormone pathways can be more or less active^14,15^. For instance, plant defense against necrotrophs or insects is generally mediated by the jasmonic acid (JA) pathway, while defense against biotrophic pathogens is mediated by the salicylic acid (SA) pathway. Several reports indicate the existence of cross-talks between those defense signaling pathways^11,12,16^. An antagonism was generally described between SA dependent defenses and JA/ET dependent defenses^17–20^. The fact that the activation of one of them represses the other suggests that plants are able to prioritize the signaling pathway they activate upon infection^16^. Interestingly, synergism between SA and JA pathways was also described^21^.

Autophagy is an evolutionary conserved process involved in the degradation of unwanted cell material^22–24^. Although the main mechanism by which autophagy contributes to cell homeostasis is thought te be the degradation of cytoplasmic components, the involvement of autophagy in protein secretion is another emerging mechanism^25,26^. Autophagy consists in the formation of a double membrane vesicle, named autophagosome, that forms arround and encloses the cargoes to be degraded^27–29^. When cargoes are captured, autophagososmes drive them towards the lytic vacuole for degradation^27–29^.

Autophagy is tightly controlled. Autophagosomes likely target specific cargoes through their interaction with the ATG8 proteins that are lipidated and anchored to autophagosome membranes. The autophagy proteins (ATG) involved in this complex machinery were first identified in yeast^30^. Most of their orthologs were found in mammals and in plants. In plant, autophagy process was found to be compromised in all the *atg* mutants defective in the single ATG genes^23,24,31^. The ATG8 protein is encoded by nine genes in Arabidopsis and although the ATG8 protein is a key player of the autophagy core machinery, no phenotype had been reported so far for the different *atg8* single mutants isolated in Arabidopsis, possibly due to functional redundancies. The conjugation of ATG8 to phosphatidyl-ethanolamine (PE) relies on a complex conjugation system that involves the ATG5 protein. The ATG2 protein is involved in lipid recruitment for autophagosome membrane elongation. Both ATG5 and ATG2 are encoded by single genes and their mutants display strong senescence and limited-growth phenotypes although phenotypes are more severe in *atg2* than in *atg5*^32–35^. Autophagy is post-transcriptionally regulated by the TOR protein kinase (Target of Rapamycin^36–38^). Autophagy genes are up-regulated under stress conditions, amongst which plant infection by pathogens^39,40^.

In plants, recent studies show that autophagy is involved in plant pathogen interactions and the involvement of autophagy machinery in disease/resistance is highly dependent on the pathosystems as well as on the plant physiological status^41–44^. The fine tuning of the cell death related to the hypersensitive response (HR), which is a strong resistance mechanism, is altered in autophagy mutants^34,45^. In addition, several studies show that compromising autophagy results in an enhanced susceptibility to necrotrophic fungi^46–48^. However it remains unclear if alteration in plant immunity is directly due to the lack of autophagy degradation process or results from indirect effects of autophagy alteration.

*Dickeya dadantii* is a necrotrophic plant pathogenic bacterium that causes soft rot disease on a large host range of crops and is able to infect *Arabidopsis thaliana*^49,50^. As is is the case of many other necrotrophs, *D. dadantii* produces large amounts of plant cell wall degrading enzymes that cause soft rots of plant tissues. Several lines of defense allow the plants to limit infection by *D. dadantii* including the production of ROS via the disturbance of iron homeostasis and by the membrane located NADPH oxidase^50–53^. Several lines of evidence show that *D. dadanti* triggers JA defense pathway^50,53^.

The role of autophagy in plant tolerance to pathogens was mainly documented regarding the cell death hypersensitive response to bacteria such as *Pseudomonas* and in response to necrotrophic fungi^44,48^. So far, there is no report dealing with necrotrophic bacteria and autophagy. Here, we show that the defect of Arabidopsis autophagy mutants’ tolerance to the bacterial necrotroph *D. dadantii* is not linked to the autophagy activity per se, but is an indirect effect of impaired fine tuning of SA defense signaling.

## Materials and methods

### Plant material

Wild type accession of *Arabidopsis thaliana* Col-0 was obtained from Versailles Arabidopsis Stock Center (INRA Versailles France, http://publiclines.versailles.inra.fr/). The autophagy mutants *atg2* (SALK_076727), *atg5* (SAIL_129B07), *atg2.sid2*, *atg5.sid2* were obtained from Yoshimoto et al. and Masclaux-Daubresse et al.^54^. The *sid2* mutant was kindly provided by Pr. JP Métraux. The *pUBI::ATG8a::GFP* overexpressor Arabidopsis line was obtained from Chen et al.^55^.

### Bacterial inoculation and quantification of disease severity

Inoculation experiments were performed with the *D. dadantii 3937* strain as described in^56^. Bacteria were grown in Luria–Bertani medium. Plants used for RNA extraction were inoculated by leaf infiltration using a syringe without a needle with a bacterial suspension at 1 × 10^8^ Colony Forming Unit/mL (CFU) made up in 10 mM MgSO_4_, mock inoculated controls consisted of leaves infiltrated with 10 mM MgSO_4_. Plants used for symptom scoring were inoculated by making a small hole with a needle in the leaf limb, and then spotting 5 μL of a bacterial suspension at a density of 1 × 10^8^ CFU/mL made up in 50 mM potassium phosphate buffer (pH 7) on the top of the hole. Symptom severity scoring was performed according to the 0 to 5 severity scale described in Rigault et al.^56^ and indicated in Supplementary Figure 1. Each symptom severity on inoculated leaves is scored then an average and a standard deviation are calculated.

### Monitoring plant gene expression by qRT-PCR

RNA extractions and RT-qPCR were performed as described in Verly et al. and Aznar et al.^57,58^ Leaves were harvested 24 h post inoculation (H p.i.) and then frozen in liquid nitrogen. Total RNAs were purified with TRIzol reagent (Invitrogen, Carlsbad, CA, USA) according to the manufacturer's instructions. The total RNA concentration was determined using a NanoDrop ND‐1000 spectrophotometer (NanoDropTechnologies Inc., Wilmington, DE, USA). RNA samples were treated with Turbo DNaseI (Ambion, Saint‐Aubin, France) RNase‐free to remove any DNA contamination. A total of 1 µg of DNase‐treated RNA was reverse transcribed using the High Capacity cDNA Reverse Transcription Kit and 50 ng of random hexamers following the supplier's instructions. One microlitre of the 1:10 diluted cDNA was subjected to real‐time quantitative PCR using SYBR Green PCR Mastermix (Applied Biosystems, Foster City, CA, USA) and gene‐specific primers designed to amplify 100–150‐bp fragments from each gene of interest and the reference genes APT and Clathrin. Primer sequences used for qRT-PCR are indicated in Supplementary Table 1.

### Salicylic acid quantification

For each sample, 2 mg of dry powder were extracted with 0.8 mL of acetone/water/acetic acid (80/19/1 v:v:v). Salicylic acid stable labelled isotope used as internal standard was prepared as described in Le Roux et al.^59^. 1 ng of standard was added to each sample. The extract was vigorously shaken for 1 min, sonicated for 1 min at 25 Hz, shaken for 10 min at 10 °C in a Thermomixer (EPPENDORF, and then centrifuged at 8000*g*, 10 °C for 10 min). The supernatants were collected, and the pellets were re-extracted twice with 0.4 mL of the same extraction solution, then vigorously shaken (1 min) and sonicated (1 min; 25 Hz). After the centrifugations, the three supernatants were pooled and dried (final volume 1.6 mL).

Each dry extract was dissolved in 100 µL of acetonitrile/water (50/50 v/v), filtered, and analyzed using a Waters Acquity ultra performance liquid chromatograph coupled to a Waters Xevo Triple quadrupole mass spectrometer TQS (UPLC-ESI–MS/MS). The compounds were separated on a reverse-phase column (Uptisphere C18 UP3HDO, 100 * 2.1 mm * 3 µm particle size; Interchim, France) using a flow rate of 0.4 mL min^−1^ and a binary gradient: (A) acetic acid 0.1% in water (v/v) and (B) acetonitrile with 0.1% acetic acid, the column temperature was 40 °C, we used the following binary gradient (time, % A): (0 min, 98%), (3 min, 70%), (7.5 min, 50%), (8.5 min, 5%), (9.6 min, 0%), (13.2 min, 98%), (15.7 min, 98%).

Mass spectrometry was conducted in electrospray and Multiple Reaction Monitoring scanning mode (MRM mode), in negative ion mode. Relevant instrumental parameters were set as follows: capillary 1.5 kV (negative mode), source block and desolvation gas temperatures 130 °C and 500 °C, respectively. Nitrogen was used to assist the cone and desolvation (150 L h^−1^ and 800 L h^−1^, respectively), argon was used as the collision gas at a flow rate of 0.18 mL min^−1^.

## Results

### Arabidopsis tolerance to the bacterial necrotroph *D. dadantii* is altered in autophagy mutants but does not necessitate autophagy activity

The role of autophagy in Arabidopsis tolerance to necrotrophic phytopathogens was only reported for fungal plant pathogens (Table 1), using *atg* mutants or transgenic lines over-expressing *ATG* genes. Consistent data were obtained in all reports showing that *atg* mutants display increased susceptibility to necrotrophic fungi. It is not the case for biotrophs and hemibiotrophs for which the role of plant autophagy depends on the pathosystem^44,48^. To know whether autophagy is also involved in Arabidopsis tolerance to bacterial necrotrophs, we addressed the issue with the model bacterial necrotroph *Dickeya dadantii*^60^.

**Table 1:** List of studies about autophagy involvement in disease cause by necrotrophic fungi on Arabidopsis.

Necrotrophic pathogen	Construct name	Mutant/ox	Phenotype	Ref
*B. cinerea*	*atg5-1*	Mutant	Enhanced susceptibility	^47^
*B. cinerea*	*atg7-3*	Mutant	Enhanced susceptibility	^47^
*B. cinerea*	*atg7-2*	Mutant	Enhanced susceptibility	^47^
*B. cinerea*	*atg18a-1*	Mutant	Enhanced susceptibility	^47^
*B. cinerea*	*ATG 18a-RNAi*	Mutant	Enhanced susceptibility	^47^
*A. brassicicola*	*atg5-1*	Mutant	Enhanced susceptibility	^47^
*A. brassicicola*	*atg7-3*	Mutant	Enhanced susceptibility	^47^
*A. brassicicola*	*atg7-2*	Mutant	Enhanced susceptibility	^47^
*A. brassicicola*	*atg18a-1*	Mutant	Enhanced susceptibility	^47^
*A. brassicicola*	*ATG 18a-RNAi*	Mutant	Enhanced susceptibility	^47^
*A. brassicicola*	*atg5*	Mutant	Enhanced susceptibility	^46^
*A. brassicicola*	*atg10*	Mutant	Enhanced susceptibility	^46^
*A. brassicicola*	*atg18a-1*	Mutant	Enhanced susceptibility	^46^
*A. brassicicola*	*atg18a-2*	Mutant	Enhanced susceptibility	^46^
*A. brassicicola*	*atg5/ATG5*	Complemented mutant	Restored WT susceptibility	^46^
*A. brassicicola*	*atg10/ATG10*	Complemented mutant	Restored WT susceptibility	^46^
*B. cinerea*	*atg2*	Mutant	No effect	^65^
*Sclerotinia*	*atg7*	Mutant	No effect	^66^
*Sclerotinia*	*atg8e*	Mutant	No effect	^66^
*Sclerotinia*	*atg12*	Mutant	No effect	^66^
*B. cinerea*	*atg5*	Mutant	Enhanced susceptibility	^74^
*B. cinerea*	*atg7*	Mutant	Enhanced susceptibility	^74^
*Plectosphaerella cucumerina*	*atg5*	Mutant	Enhanced susceptibility	^46^
*Plectosphaerella cucumerina*	*atg10*	Mutant	Enhanced susceptibility	^46^
*Plectosphaerella cucumerina*	*atg5/ATG5*	Complemented mutant	Restored WT susceptibility	^46^
*Plectosphaerella cucumerina*	*atg10/ATG10*	Complemented mutant	Restored WT susceptibility	^46^

**Figure 1:**
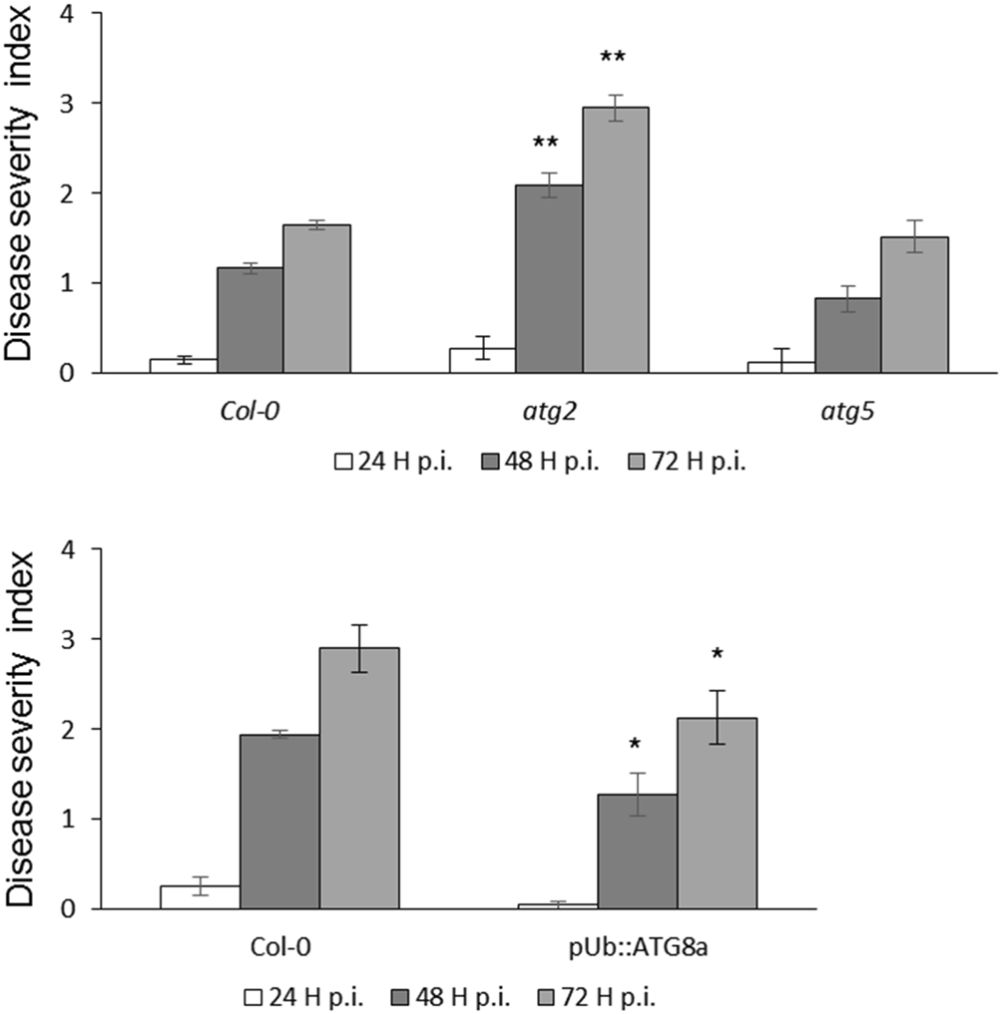
Disease severity on *atg* mutant and *ATG8a* overexpressing lines: indicated genotypes were inoculated with *D. dadantii* and disease severity was monitored over 3 days according to the severity scale^56^. Bars are standard errors. N = 60 leaves from 20 plants. **p < 0.01, *p  < 0.05 *t* test comparing means to the Col-0 WT. Experiments were performed 3 times with similar results.

For this purpose, two different *atg* mutants, one affected in lipid recruitment for autophagosome formation (*atg2*) and the other affected in the conjugation system permitting ATG8 lipidation and anchorage to the membrane of autophagosomes (*atg5*) were inoculated with *D. dadantii*. Wild type Col-0 and the two *atg2* and *atg5* mutants were inoculated by spotting a bacterial suspension on leaves of 6 weeks old plants. The severity of the symptoms was scored based on 0–5 scale^56^. Severity of the symptoms on *atg2* mutant was higher over time than that observed on the WT plants (Fig. 1). Interestingly, the susceptibility level of the *atg5* mutant was similar to that of the WT. The absence of difference between *atg5* mutant and WT was confirmed on the two *atg5* mutant allele (*atg5-1* and *atg5-2*^33^; data not shown). This indicates that only *atg2* was more susceptible than Col-0 to *D. dadantii* while the *atg5* mutant known to display a less severe yellowing phenotype than *atg2*, was not affected in its susceptibility to *D. dadantii.* Such difference between *atg2* and *atg5*, then raised the question of the role of the autophagy machinery in the tolerance to *D. dadantii*, and the potential link with a senecence status of the mutants.

We then used an Arabidopsis line with enhanced autophagic activity consisting in the overexpression of the ATG8a protein under the control of Ubiquitin promoter^55^. Following *D. dadantii* inoculation, plants overexpressing *ATG8a* displayed reduced symptom severity compared to the WT suggesting a positive role of autophagy activity on the tolerance of Arabidopsis to *D. dadantii* (Fig. 1).

### Autophagy genes are not up-regulated upon *D. dadantii* infection

**Figure 2:**
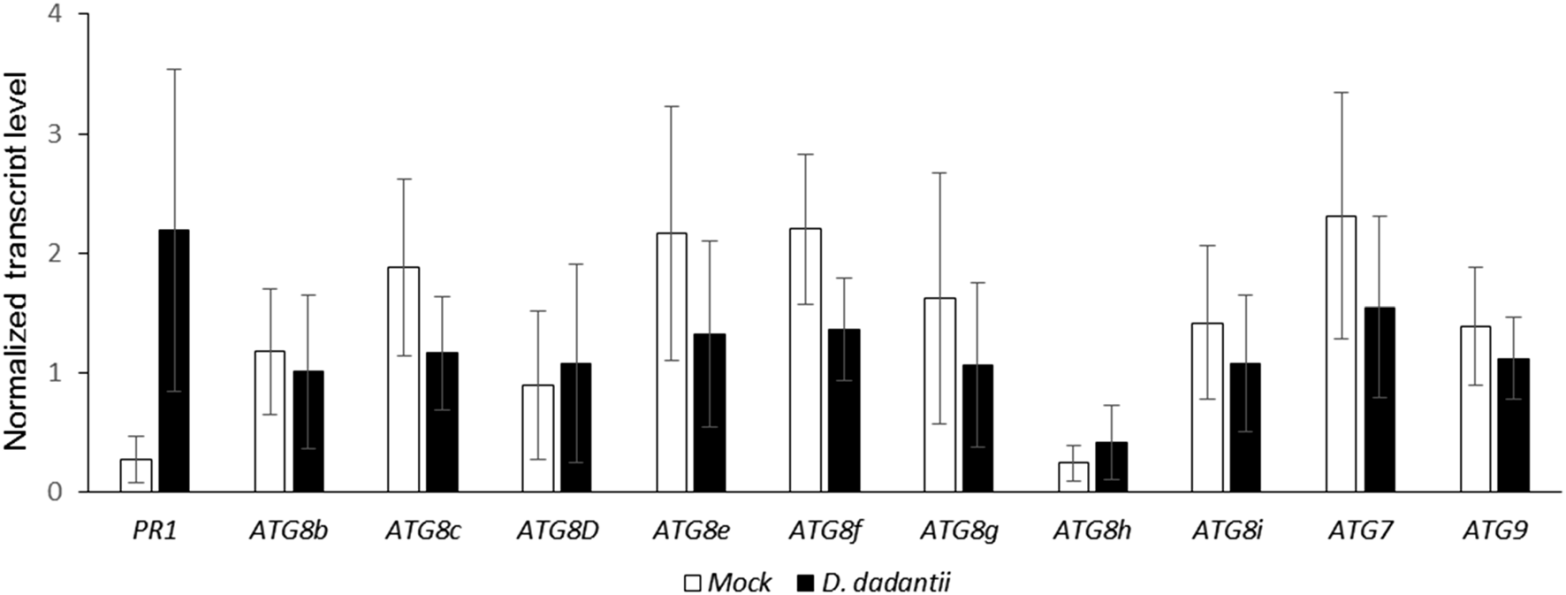
Expression of *ATG* genes upon *D. dadantii* infection. Plants were infected or mock treated then leaves were harvested 24 h after treatment. Transcript levels were monitored by qRT-PCR and normalized against the transcripts of the reference genes *APT and Clathrin.* Bars are standard deviation, N = 4. Experiments were performed 3 times with similar results.

As disease symptoms observed on the different *atg* lines suggested a link between plant response to *D. dadantii* and autophagy, we monitored *ATG* gene expression following plant inoculation. RT-qPCR was performed to monitor the transcript level of 10 autophagy genes chosen for their positive response to stress in litterature^40^. None of these genes was found to be upregulated in response to infection (Fig. 2) by contrast with the defense gene marker *PR1* that was upregulated in the same plants as expected. These data suggest that transcriptional activation of autophagy genes was not required in the response of Arabidopsis to *D. dadantii* infection.

### Salicylic acid dependent susceptibility of *atg2* mutant to *D. dadantii*

Our results indicate that functional ATG2 protein is essential to control disease symptoms in Arabidopsis upon *D. dadantii* infection but that working ATG5 protein and operational autophagy is not essential. Even though autophagy genes are not induced by infection, enhanced constitutive autophagic activity provides positive effect on plant tolerance to *D. dadantii*. If autophagy machinery was directly involved in Arabidopsis tolerance to the bacterium, then any knock-out mutant affected in autophagy should be more susceptible to the bacterium*.* Our results suggest that defect in autophagy activity was not the direct cause of Arabidopsis susceptibility to *D. dadantii.* Indirect effects of autophagy defects on plant tolerance to pathogens might be related to hormonal balance. It is well known that autophagy defect triggers hormonal disorders and especially exacerbates SA production and SA signaling, then enhancing fast and spectacular leaf senescence like symptoms^41,42^. Because senescence/yellowing phenotypes observed on the *atg2* mutant is stronger than in the *atg5* mutant, we suspected exacerbation of SA response in *atg2* by comparison with *atg5* and considered the possibility that SA accumulation in *atg2* was the origin of its enhanced susceptibility to *D. dadantii.*

**Figure 3:**
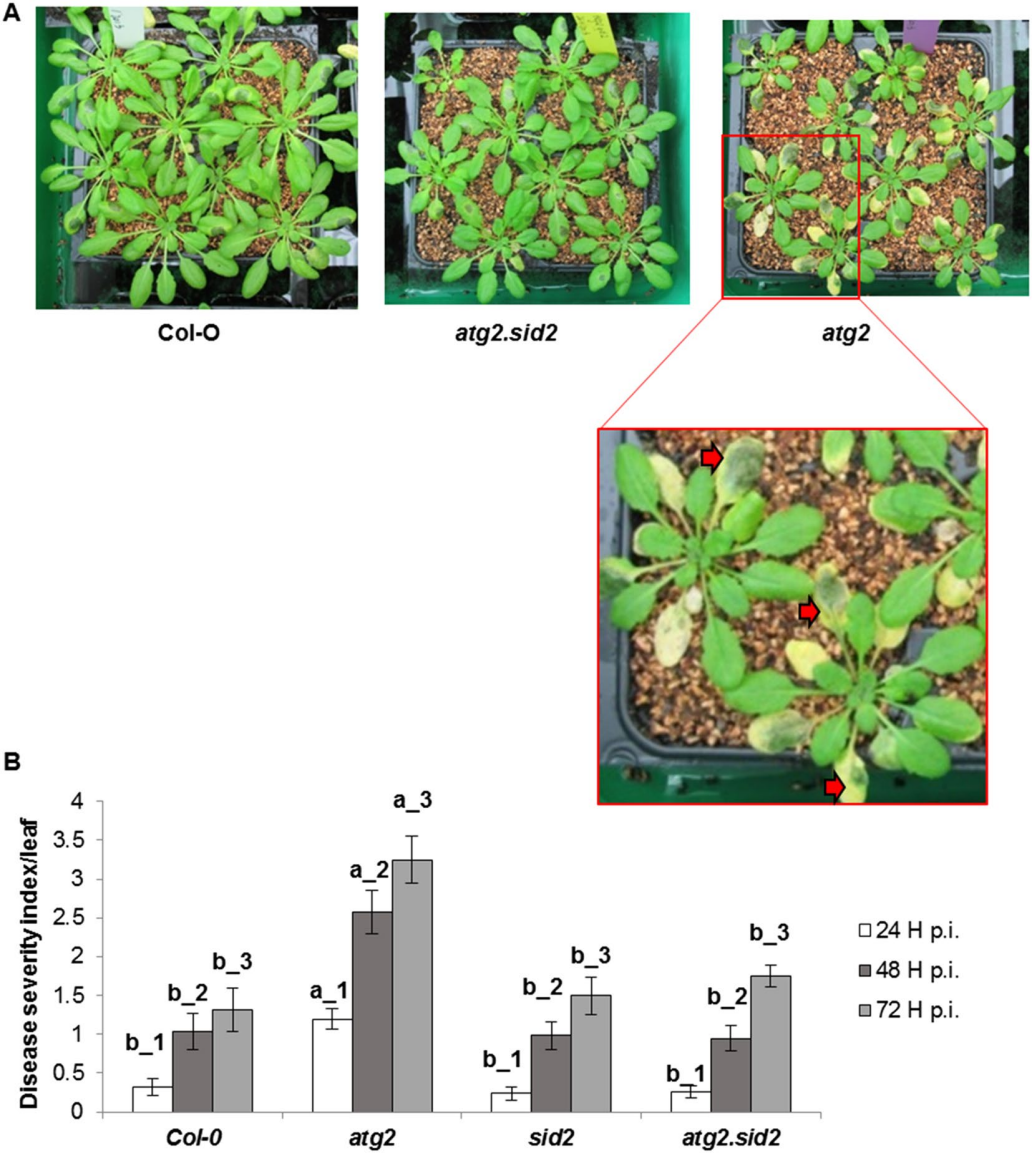
Susceptibility of *atg2* mutant relies on SA. (**A**) Pictures of 6 week-old Arabidopsis indicated genotypes inoculated with *D. dadantii.* In the enlargement of *atg2* mutant inoculated plants, red arrows indicate yellow zones of early senesce. (**B**) Symptom severity at indicated time points on indicated genotypes. Bars represent standard deviation. N = 30. Experiments were performed three times with similar results. Different letters in the graph indicate statistical significance between genotypes at the same time point (one-way ANOVA with Tuckey’s test; P < 0.05).

In order to determine whether SA is involved in the increased susceptibility of *atg2* mutant, we monitored the susceptibility of the *atg2.sid2* double mutant defective in SA synthesis as the *SID2* gene encodes an isochorismate synthase which is involved in SA biosynthesis in Arabidopsis, in particular in response to pathogens^61^. Figure 3A shows the phenotypes of Col-0 wild type, *atg2* and *atg2.sid2* mutants infected with *D. dadantii*. Both soft rot symptoms and leaf yellowing phenotypes were clearly more severe on *atg2* leaves than on Col-0 and *atg2.sid2* leaves (Fig. 3). Although *atg2* was early senescing by comparison with Col-0 and *atg2.sid2* plants^34^ (Fig. 3A), we observed enhanced chlorosis around the *D. dadantii* inoculation spots which were not present in uninfected plants or before inoculation (data not shown). This suggested that infection accelerated leaf senescence symptoms on *atg2* plants. It can be noticed that disease severity was similar in WT, *sid2* and *atg2.sid2* double mutant. These data indicate that the higher susceptibility of the *atg2* mutant can be restored to the WT level when *SID2* gene is non-functional, suggesting the prominent role of SA in the increased susceptibility of *atg2* mutant to *D. dadantii*. This confirms that autophagy degradation pathway is not essential for Arabidopsis tolerance to *D. dadantii*. Nevertheless, higher constitutive autophagic activity artificially enhanced by *ATG8a* overexpression may help increasing plant tolerance, possibly through positive effects on leaf longevity, meaning negative effect on SA production.

### Salicylic acid signaling rather than SA amount is involved in *atg2* mutant enhanced susceptibility to *D. dadantii*

Previous reports indicate that the level of salicylic acid in autophagy mutants is higher than that of WT^54,62^. To determine the mechanism by which high SA level could lead to enhanced susceptibility, we hypothesized that high amounts of SA could repress the JA signaling pathway which is known to limit *D. dadantii* infection^50,57^. Indeed, it is commonly reported that SA signaling represses JA signaling and that ET and JA signaling are commonly synergistic^17–20,63^.

**Figure 4:**
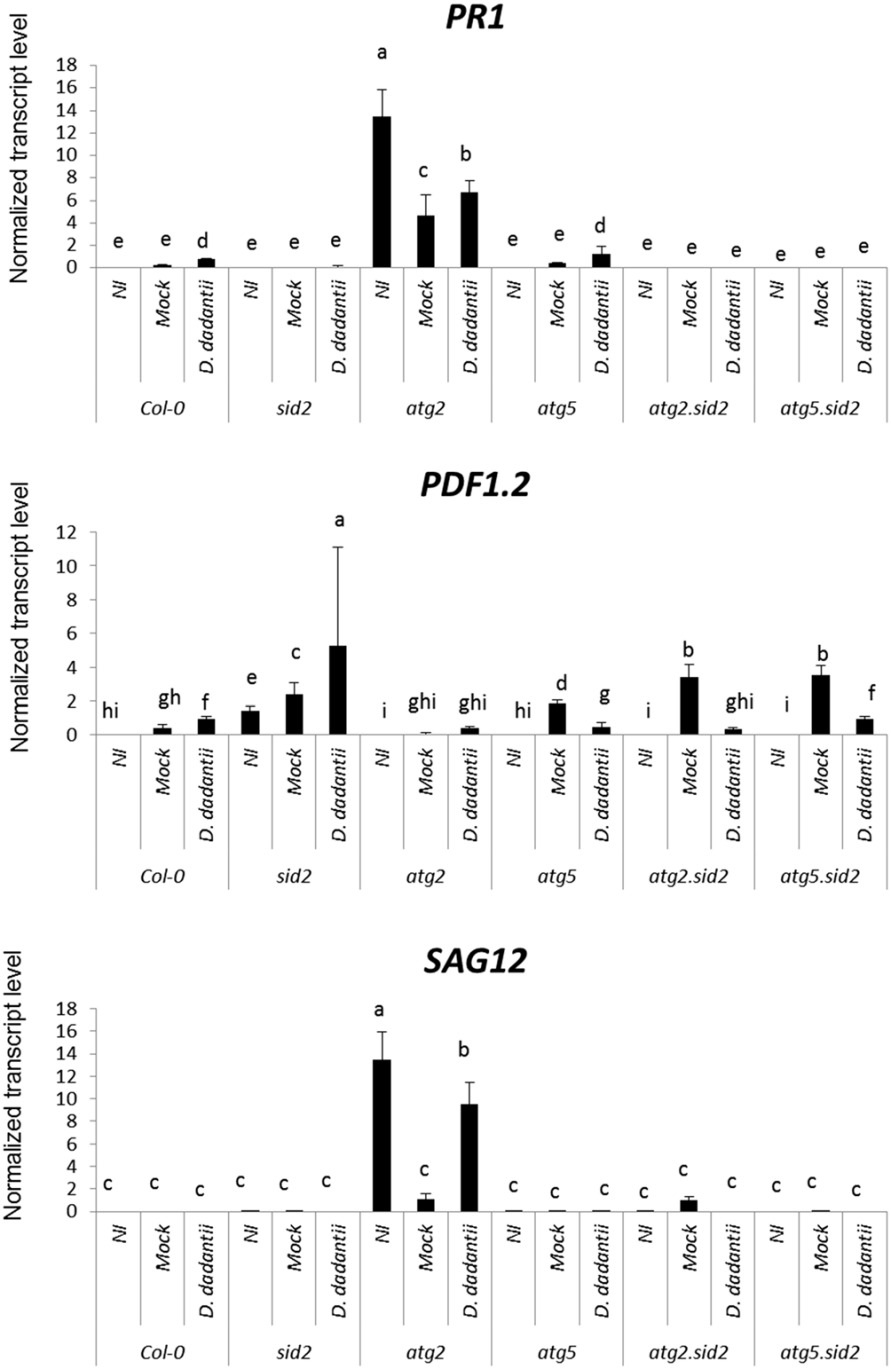
Expression of defense and senescence genes upon *D. dadantii* infection. Plants were infected or mock treated, leaves were harvested 24 h after treatment. Transcript levels were monitored by qRT-PCR and normalized against the transcripts of the reference genes *APT and Clathrin*. Bars represent standard deviation. N = 4. Experiments were performed three times with similar results. Different letters in the graph indicate statistical significance between treatments (one-way ANOVA with Tuckey’s test; P < 0.05). NI: Non- inoculated, Mock: treated with MgSO_4_.

To further investigate the possibility that SA and JA signaling were modified in *atg2* mutants, we determined the status of *SA* and *JA* mediated defenses in *Col-0*, *sid2*, *atg2*, *atg5*, *atg2.sid2* and *atg5.sid2* plants. For this purpose, we monitored the expression of defense marker genes of the SA (*PR1*) and the JA (*PDF1.2*) pathways in infected plants. Controls consisted of plants inoculated with 10 mM MgSO_4_ and naïve plants that were untreated. The latter naïve plants were used to determine the initial immunity level of plants before inoculation. Transcript levels of both the SA defense marker gene *PR1* and of the JA defense marker gene *PDF1.2* were increased in WT Col-0 plants following *D. dadantii* infection compared to controls (Fig. 4). While no *PR1* transcript was detected in the *sid2* mutant line, higher levels of *PDF1.2* transcripts were detected in these plants compared to Col-0 confirming the repressive action of the SA pathway on the JA pathway. Strikingly, in the *atg2* mutant, higher levels of *PR1* transcripts were detected compared to the WT (10 times higher in *atg2* compared to Col-0 infected plants) and an up-regulation of this level was observed in response to *D. dadantii* compared to the mock inoculated plants. The level of *PR1* transcripts in *atg5* mutant was unchanged compared to the WT indicating that SA signaling in the *atg5* mutant was not affected. The expression level of *PDF1.2* was reduced in the *atg2* mutant compared to that of Col-0 which is consistent with a high SA signaling. The transcript level of *PR1* was undetectable in both *atg2.sid2* and *atg5.sid2* double mutants, indicating that SA signaling was totally abolished by introducing the *sid2* mutation. Interestingly, in naïve *atg* simple and double mutant plants, the level of *PDF1.2* transcripts was reduced compared to that of mock inoculated plants. Our data suggest that mechanical stress triggers JA signaling in *atg* mutant backgrounds, a process which is repressed by *D. dadantii* infection. To know whether senescence was also involved in this interaction, the transcript level of the senescence marker gene *SAG12* was monitored. *SAG12* was only expressed in *atg2* mutant plants (Fig. 4). Intriguingly, it was down regulated by mock inoculation and up-regulated by infection confirming the accelerated senescing phenotype we observed on *atg2* infected plants compared to mock *atg2* plants.

**Figure 5:**
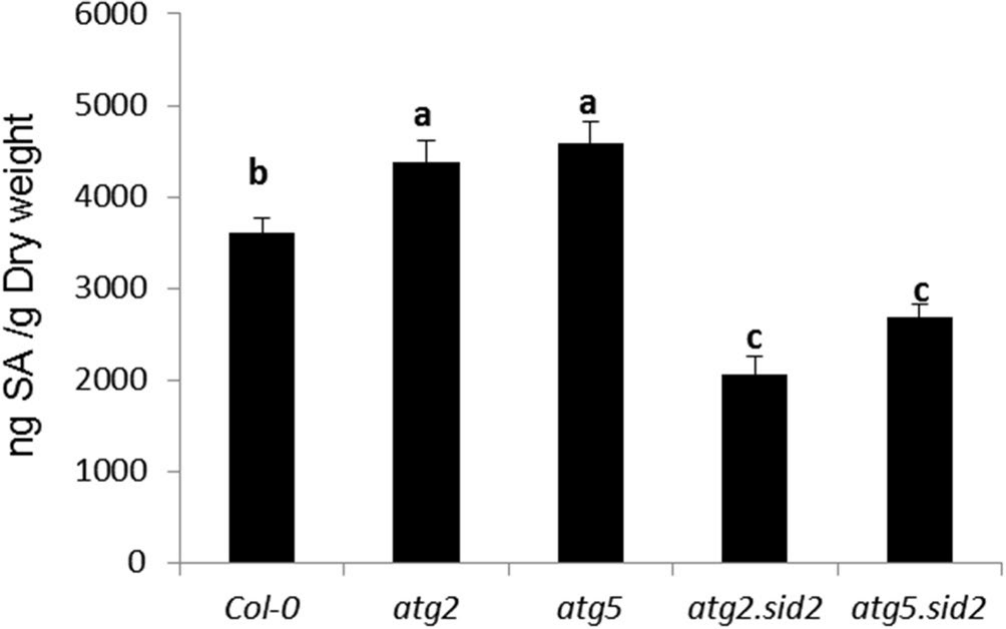
Salicylic acid content in *atg* mutants: Six week old plants of indicated genotypes were harvested then SA content was monitored as indicated in Materials and Methods. Bars represent standard deviation. N = 10 to 12. Experiments were performed three times with similar results. Different letters in the graph indicate statistical significance between treatments (one-way ANOVA with Tuckey’s test; *P* < 0.05).

To investigate the role of SA content in the response of autophagy mutants to *D. dadantii*, SA content was quantified in naïve plants (Fig. 5). Both *atg2* and *atg5* mutants accumulate higher SA levels than Col-0 plants. The level of SA was reduced in both double mutants *atg2.sid2* and *atg5.sid2* relative to single mutants confirming the role of *SID2* in SA production in Arabidopsis.

## Discussion

The mechanisms by which plant autophagy affects immunity are complex and vary depending on the considered pathosystems. In some cases, opposite mechanisms can take place depending on plant age or infectious stage. For example, in young Arabidopsis leaves, autophagy has a pro-death activity enhancing HR mediated cell death^45^ while in older leaves, autophagy seems to counteract cell death^34^. Plant autophagy was shown to counteract the bacterial pathogen *Pseudomonas syringae* pv. *tomato* which, surprisingly, secretes an effector able to trigger autophagy^64^.

Several reports indicate that the susceptibility of *atg* mutants was increased in response to fungal necrotrophic pathogens (Table 1). To determine the effect of *atg* mutations on Arabidopsis susceptibility to the bacterial necrotroph *D*. *dadantiii*, we used an *atg2* and *atg5* mutants. The *ATG2* and *ATG5* genes are single genes in Arabidopsis as in many plant species, and are essential for autophagic activity as their mutants cannot form autophagosomes. The *atg2* and *atg5* mutants were previously characterized by many groups amongst which Pr. R. Vierstra and Pr. K. Yoshimoto groups, and display the typical hypersensitivity to abiotic stresses described for many autophagy mutants in Arabidopsis^33,34^ that is related to leaf yellowing phenotypes and necrotic spots. It might be noticed that *atg2* mutant phenotypes are stronger than those of *atg5* mutant. Our findings showing different behaviors of two autophagy mutants are not surprising with regard to the literature. Indeed, it appears from the literature that the susceptibility of *atg* mutants to different pathogens is not always similarly affected compared to the WT plants. For instance, although we found that in response to *D. dadantii*, the *atg2* mutant showed enhanced susceptibility, the susceptibility of the same *atg2* mutant to the necrotrophic fungus *Botrytis cinerea* has not been found affected^65^. In *atg7 a*nd *atg12* Arabidopsis mutants, the susceptibility to the necrotrophic fungus *Sclerotinia sclerotiorum* was shown unaffected^66^. All these reports indicate that although a general trend observed in autophagy mutants is the susceptibility to necrotrophic pathogens, some mutants are likely not affected.

A common trend in necrotrophy is cell wall degrading enzyme production by pathogens. Cell wall homeostasis is tightly linked to membrane stability which could be in part under the control of autophagy^29,67,68^. The mechanisms explaining the higher susceptibility of some *atg* mutants to necrotrophic pathogens remained unknown^44,48^. One hypothesis is that as a pro-survival mechanism, autophagy hampers the growth and development of necrotrophs which proliferate preferentially on dead cells. In this situation, autophagy would ensure the clearance of degraded cellular components thus protecting plant tissues from activating stress responses such as the SA signaling pathway^44^. Another hypothesis is that the hormonal balance which is disturbed in *atg* mutants favors necrotrophs. Both hypotheses are not mutually exclusive and can depend upon the the pathosystem considered. In the interaction between Arabidopsis and *D. dadantii*, we demonstrate that the enhanced susceptibility of the *atg2* mutant relies on SA signaling Our data also show that the senescing state of the plants correlates with disease development since *SAG12* expression and chlorotic phenotype were enhanced in *atg2* plants and lost in the *atg2.sid2* mutant. It is however intriguing that there is no correlation between SA content and *PR1* expression level in *atg2* and *atg5* mutants. One interpretation is that senescence and SA form an amplification loop in *atg2* and that both are required to trigger high susceptibility^34^. Such amplification loop between senescence and SA was indeed recently described^69,70^.

We showed that in *atg2* higher SA content and signaling coincided with low JA signaling thus explaining increased susceptibility, as JA signaling has been shown to contribute to Arabidopsis tolerance to *D. dadantii*^50,57^. The disease phenotype of the *atg5* mutant, which is similar to that of the WT plants, may be explained by the fact that *atg5* is senescing later than *atg2* mutant. This may be due to a differentially active senescence and immunity molecular machinery in each *atg* mutant. For instance, transcript levels of genes encoding transcription factors related to immunity and senescence (WRKY, ERF and JAZ) are different in an *atg5* compared to an *atg9* mutant^54^. Interestingly, ATG9 interacts with ATG2 in the phagophore expansion process^23^ suggesting that *atg9* and *atg2* mutants could harbor the similar immunity and senescence defects that differ from those of the *atg5* mutant. In addition to the differential expression of transcription factor encoding genes in different *atg* mutants, it is possible that different proteolytic activities reside in each mutant. Indeed, it was recently shown that autophagy deficiency in the *atg5* mutant resulted in an alteration of cellular proteolytic activities^67^. The actors of immunity including transcription factors may be targeted by proteolysis which ensures fine tuning of adequate defense responses^71,72^. It would be interesting to compare the proteolytic activities in *atg2* and *atg5* mutants to investigate the lifetime of key immunity and senescence related transcription factors.

Altogether, we provide here the evidence that SA plays a pivotal role in the enhanced susceptibility of *atg2* mutant to *D. dadantii* through the repression of JA-dependent defenses*,* and that autophagy degradation function is not directly involved in plant tolerance to *D. dadantii*. While our study argues against the involvement of plant autophagy in the tolerance to this type of aggressors, it confirms the importance of leaf senescence status in the susceptibility of plants and points to the hormonal balance as a key player in this process. In addition, overexpression of ATG8 proteins, that had been shown to provide positive effects on plant tolerance to many stresses such as drought in several plant species (Chen et al.^73^ for a review), significantly increased the tolerance of our Arabidopsis plants to *D. dadantii* infection.

## Supplementary Information

Supplementary Figure S1.

Supplementary Table S1.
