# Supplementary material for: Salicylic acid is a key player of Arabidopsis autophagy mutant susceptibility to the necrotrophic bacterium Dickeya dadantii
Source: Sci Rep. 2021 Feb 11;11:3624. doi: 10.1038/s41598-021-83067-6 (PMC7878789; doi:10.1038/s41598-021-83067-6)
Supplement: Supplementary file 1 — Supplementary Figure S1. [file 41598_2021_83067_MOESM1_ESM.pptx]

## Slide 1
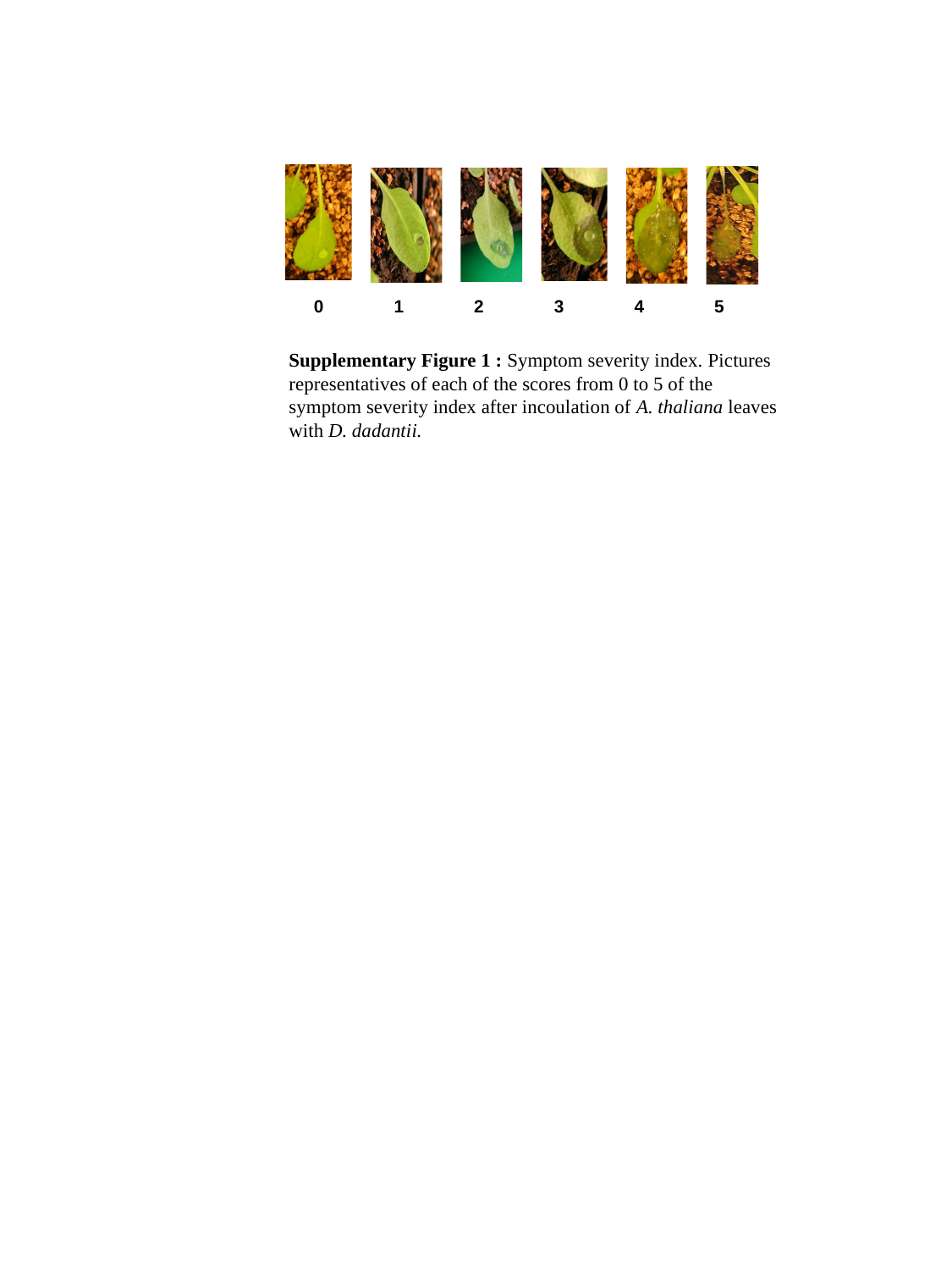

0
1
2
3
4
5
Supplementary Figure 1 : Symptom severity index. Pictures representatives of each of the scores from 0 to 5 of the symptom severity index after incoulation of A. thaliana leaves with D. dadantii.
